# Sigmoid volvulus part 1: Understanding patient experiences and priorities

**DOI:** 10.1111/codi.70520

**Published:** 2026-06-16

**Authors:** Sue Blackwell, Zak Shehata, Carly Bisset, Jennifer Law, Hwei Jene Ng, Susan Moug, Nick Heywood

**Affiliations:** ^1^ Institute of Applied Health Research University of Birmingham Birmingham UK; ^2^ North West Deanery Manchester UK; ^3^ Department of Surgery NHS Greater Glasgow and Clyde, Glasgow Royal Infirmary Glasgow UK; ^4^ Blackpool Teaching Hospitals NHS Trust Blackpool UK; ^5^ Royal Alexandra Hospital University of Glasgow Paisley UK; ^6^ East Lancashire Hospitals NHS Trust University of Central Lancashire Blackburn UK

**Keywords:** patient priorities, sigmoid volvulus

## Abstract

**Aim:**

Sigmoid volvulus is one of the commonest presentations of bowel obstruction worldwide but remains underresearched. We aimed to discover patients' priorities in treatment options for sigmoid volvulus, and how management strategies impact their quality of life.

**Methods:**

Patients and/or their relatives/carers who had been diagnosed with sigmoid volvulus in the last 5 years were invited to attend a Patient and Public Involvement (PPI) event. Small group and whole group facilitated discussion focused on patients' experiences, priorities and outcomes. Discussions were facilitated by colorectal consultants and surgical trainees, as well as a patient researcher.

**Results:**

Six patients and three relatives/carers attended the PPI event, along with a patient researcher, four surgical trainees and two consultant colorectal surgeons. Identified themes which future studies would need to consider as patient related outcome measures were: quality of life, patient and carer anxiety, communication, decision‐making, recovery and the impact on carers. Patient experience was highly varied and included recurrent episodes requiring endoscopic detorsion and undergoing emergency or elective surgery. In future studies, participants would recommend the use of qualitative interviews and inclusion of patients who had died from complications of sigmoid volvulus, explored through qualitative interviews with partners and carers.

**Conclusion:**

The patient and their relative/carer's experience of sigmoid volvulus is highly variable and currently underexplored. A holistic approach to treatment with shared decision‐making between the patient, their carer and their surgeon is essential.

**What does this paper add to the literature?**

This paper explores the patient and carer perspective of sigmoid volvulus, something that hitherto has been underreported in the literature. This paper highlights that the patient and their relative/carer's experience of sigmoid volvulus is highly variable and a holistic approach to treatment with shared decision‐making between patient, their carer and their surgeon is essential.

## INTRODUCTION

Sigmoid volvulus is a common emergency surgical presentation responsible for 10–15% of large bowel obstructions in the western world [1]. Volvulus most commonly occurs in men over 70 years of age, with a male/female ratio of 4:1. Risk factors include a high fibre diet, chronic constipation, frequent laxative use, as well as neuropsychiatric disorders. The incidence of sigmoid volvulus is much greater in Africa, South America, Russia and the Middle East, with incidence ranging from 13 to 42%; however, this often affects a different patient group than in the Western World [2, 3, 4].

The sigmoid colon can become dilated and faecally loaded, making it vulnerable to volvulus, most commonly counterclockwise [5]. After multiple episodes, the mesentery shortens secondary to chronic inflammation. This can then lead to adhesion formation, which, if it compromises the vascular pedicle, can result in ischaemia.

The common symptoms of sigmoid volvulus are that of large bowel obstruction–abdominal distension, abdominal pain, absolute constipation, nausea and vomiting [6]. While a plain abdominal film can diagnose sigmoid volvulus, a contrast enhanced CT is the gold standard to both diagnose and assess bowel viability [6].

In the emergency setting, sigmoid volvulus should be diagnosed promptly and decompressed via either flexible or rigid sigmoidoscopy to achieve decompression and luminal visualisation [6]. If the patient already has peritonism, or flexible sigmoidoscopy is unsuccessful, then an operative approach is recommended. The Association of Coloproctology of Great Britain and Ireland recommends that the optimal treatment for sigmoid volvulus is resection of the affected colon. While it is the definitive management for sigmoid volvulus, surgery often poses significant risk in this cohort of patients, many of whom are older frail patients with multi‐morbidity. Such patients are often managed conservatively with endoscopic decompression; however, this carries a high recurrence rate that has been reported as high as 54% [7]. This can lead to repeated hospital attendances, which is not only extremely debilitating and affects quality of life, but also puts pressure on hospital services. Although other potential non‐surgical options include percutaneous endoscopic colostomy (PEC), the evidence base for its use is limited, as is the expertise, and like primary decompression carries risks including bowel perforation [8].

There is significant variability worldwide in the management of sigmoid volvulus, with no standardised approach regarding the timing and method of surgical management. The existing literature has a distinct lack of controlled trials and comprises large case series or single centre studies, with inadequate follow up and lack of reporting of patient outcomes [9, 10, 11].

Furthermore, there is a lack of published literature focusing on the patient experience for those affected with sigmoid volvulus and no standardised outcome measures. RESoLVE: REview of Sigmoid VoLVulus ManagEment (RESoLVE) is a research study that aims to explore all areas of management of sigmoid volvulus, starting with a multi‐centre prospective audit. As part of the exploratory work for the multi‐centre audit, we aimed to gather patient and carer views on current management of sigmoid volvulus and the impact on quality of life to help understand what is important to patients and carers, with the intention that this would help to inform the design of future elements of the study.

## METHODS

A Patient and Public Involvement (PPI) event was held in October 2023. This was a novel hybrid PPI discussion across two sites (Scotland and England) where in‐person facilitators were available at both sites, which were linked together through an online meeting. It was supported with funding from Bowel Research UK, in line with NIHR UK Public Involvement Standards [12]. Outcomes from the PPI day are reported according to the GRIPP2SF checklist [13]. No ethical approval was required as per the HRA guidance [14]. This paper reports the events of the PPI day.

### PPI inclusion

Patients, and relatives/carers of patients, who had been diagnosed with sigmoid volvulus in the last 5 years were invited to attend. Participants were identified through recent clinical input and were invited to take part. A patient information sheet and invitation letter were produced in conjunction with the patient researcher and sent to interested patients to inform them about what to expect on the day. All patients approached were able to travel to the meeting, and travel expenses were reimbursed.

### Study team

Members of the RESoLVE study team were invited to attend. These included a patient researcher, colorectal surgeons and general surgical trainees.

### Outline of PPI day

The aim of the PPI day was to establish patient perspectives around their experience and priorities in relation to treatment options for sigmoid volvulus, and how management strategies impact their quality of life, to help inform further elements of the RESoLVE study. Small group and whole group facilitated discussion focused on patient experiences, priorities and outcomes. A visual presentation explained the outline of the PPI day. In order to allow a wide range of patients to participate, Microsoft Teams was utilised to allow a live video link between two sites. Both sites had colorectal consultants and surgical trainees in attendance. Each site discussed the questions (personal experience of sigmoid volvulus, feedback on proposed audit, what was important to cover in future research) and then lead facilitators from each site reported back to the wider group on the emerging themes and a facilitated full group discussion was undertaken.

## RESULTS

### PPI characteristics

Six patients and three family members participated in the PPI day (age range 55–75) (Table 1). Seven were male, two female. All six patients had undergone surgery for sigmoid volvulus. Four were operated on during their index presentation, one had recurrent admissions and had elected for surgery, and one had recurrent episodes of sigmoid volvulus managed at home and subsequently underwent an emergency operation. Two of the family members were husband and wife, who were representing their daughter (A) with Cerebral Palsy, and who had experienced recurrent episodes of sigmoid volvulus, but who was yet to undergo any surgery. One spouse and one patient who were unable to attend sent in a letter recounting their experience.

**TABLE 1:** Demographics of patients attending PPI day.

Identifier	Sex	Age	Sigmoid volvulus history	Surgery	Notes
Patient A	Female	30	Recurrent episodes	None	Represented by parents
Patient B	Male	75	Recurrent episodes	Elective	
Patient C	Male	57	First presentation	Emergency	
Patient D	Female	55	Recurrent episodes	Emergency	Accompanied by husband
Patient E	Male	71	First presentation	Emergency	Wife sent letter
Patient F	Male	65	First presentation	Emergency	
Patient G	Male	68	First presentation	Emergency	
Patient H	Female	83	Recurrent episodes	None	Sent letter unable to attend

Seven members of the study team were in attendance: consultant colorectal surgeons (2), general surgery trainees (4); patient researcher (1).

### Analysis

Notes were taken by members of the study team. Six themes were identified through the discussion and agreed with the group at the PPI event. Post event analysis following thematic analysis was undertaken to check that identified themes were appropriately described [15]. No changes were made to thematic labels at this stage. Notes were typed up and quotes were selected as representative of the group discussions for each theme.

#### Quality of life

The impact on the quality of life of both the patients and their partners and relatives was one of the main themes identified. Repeated admissions to hospital significantly affected the quality of life of patients and in some cases had guided their decision to undergo surgical intervention. Some participants reported that the lack of community support from their GP and lack of widespread knowledge of the condition had a negative impact on their quality of life, as often community care does not have the capacity to provide intensive preventative care, particularly in the approach to constipation.

“*Accepted it will come back eventually… may not make it, I'm 83*” Patient H.

#### Patient & relative/carer anxiety

Anxiety around when an episode of sigmoid volvulus would recur was of importance to both the patients and their family members. The uncertainty and the subsequent impact on planning aspects of everyday life were raised by all the group members.

“*Once we got to A&E I started to feel safe*” Patient C.

Patient A's parents described the ‘*fear that her bowel is going to burst*’ when discussing the difficulties in deciding when she needs hospital treatment or not. It was clear that there was additional anxiety around being a carer and having to make decisions for their loved one when their relative lacked capacity. It was agreed that future research should include impact on relatives and carers.

#### Communication

Patients experienced the effects of poor communication between hospital and community for aftercare, with a feeling that instructions from the hospital were not being followed by primary care services. Due to the acute nature of the problem, particularly in the first presentation, some patients were unaware of the possible risks of the major surgery they had previously undergone. Communication in the acute and elective setting was very different, with the elective setting providing more opportunity to consider a variety of options.

Communication between the surgical team and patients and their relatives was highlighted as an important area; where surgeons had taken the time to explain what was happening, it was reassuring to both patients and carers.

“*he explained clearly what was happening, and what could be the possible outcomes of the op*” Patient E.

#### Decision‐making

Decision‐making was an area that was important to all the group members. For those patients that had undergone emergency surgery, they reported there was little or no decision‐making to be done as there was only one clear option in their mind: surgery. In contrast, those who had undergone elective surgery stated they had needed to make decisions about when to elect for surgery and consider the impact that would have on their quality of life and that of others. However, it was generally felt that the individuals looked to the surgeon for help making the right decision for them.

*“ask the surgeon to do what's best for me, it's a difficult question to answer – I haven't got the skills to make that decision”* Patient D.

One patient who had undergone elective surgery felt that the current treatment escalation, that is starting with decompression and then moving to elective surgery if there was a recurrence was better than going straight for surgery. He preferred the cautious approach as this gave him time to consider his options, especially as he was aware that surgery may result in a stoma

“*no one wants a stoma unless they have to*” Patient B.

One of the participants spoke about the differences between him and his wife in regard to their perception of risk, and this was important when he was making decisions about surgery. He felt that his job had made him more willing to take risks than his wife would potentially consider

“*my wife worries about every little thing, whereas I think well, whatever happens, will happen*” Patient B.

For those who were carers, the burden of decision‐making was high. Patient A's parents highlighted that it can be difficult for her professional carers to decide about when to take her to hospital. Her admissions are frequent, and disrupt both her life and theirs. They were at the point where some form of surgical intervention was necessary, to give both her, and them, back some quality of life. They highlighted the difficulties around what operation was best; what the side effects would be, would she survive surgery with her comorbidities and that surgery may make things worse rather than better.

#### Recovery

Recovery from both episodes of sigmoid volvulus, and surgery for it, was an area that all of the participants felt was important to evaluate. Those who had experienced recurrent episodes of sigmoid volvulus reported that, while recovery from endoscopic intervention was quick, the repeated episodes had a cumulative effect and impacted recovery. Those that had undergone surgical intervention had different recovery concerns, such as getting used to a stoma or learning to live with altered bowel function due to the extent of colon that was resected.

“*It's early days but I can't believe how good I am (after surgery)*” Patient G.

#### Impact on family members

The family members present, as well as many of the patients, felt that the experience of sigmoid volvulus was a ‘shared journey’. The patient who had elected for surgical intervention said that they hadn't realised how much it affected their partner, in terms of being able to work or carry out normal duties due to caring for their loved one.

The group members who were carers highlighted the impact that their family members' illness had on them. Patient A's parents talked about how they had to plan their lives around her admissions. While she lives in supported living accommodation, they wanted to be with her when she was admitted to hospital which would mean dropping all activities such as returning home from holiday if they were away at the time. A personalised admission plan had been put in place and she had been granted direct access to the surgical ward, which had vastly reduced her length of stay and had allowed them to be able to live a more normal life, despite repeated admissions with sigmoid volvulus.

#### Potential for further research on sigmoid volvulus

The group discussed the possibility of future research on sigmoid volvulus. This work would follow on from the prospective observational cohort study that had been planned. All agreed that this was an area that needed further research and that any future research must include both patient/carer and clinician‐reported outcomes.

The group felt that any future research should also include patients who had died from complications of sigmoid volvulus, which could be explored through qualitative interviews with partners and carers. They felt that this was important to fully understand the impact of sigmoid volvulus.

## DISCUSSION

We believe that this is the first time that patients who have experienced sigmoid volvulus and their family/carers have been asked about their experiences of the condition and the treatment options.

The main themes identified were that of the impact of volvulus on quality of life, as well as the anxiety regarding recurrent episodes compared with the anxiety of major surgery. Although endoscopic therapy can be an alternative to the significant undertaking of major surgery, the poor access to tailored emergency care for decompression is a major upheaval for patients. This is further compounded by patients' mobility and level of understanding, where recurring trips to hospital are difficult and distressing.

This study highlights that each case of sigmoid volvulus affects patients in different ways and that measuring outcomes of larger scale research studies can be challenging due to the heterogeneity of the patient cohort. Some are affected by life changing emergency surgery, while others face the regular upheaval of recurrent episodes and multiple hospital admissions. Of those who did not require emergency surgery, the concept of decisional regret was apparent, not just for the patient but for the families and carers as well. This should be an aspect that could be explored through patient questionnaires or qualitative interviews. Further qualitative work is needed to explore if patients in populations with a higher prevalence of sigmoid volvulus have similar experiences and priorities as those identified by our patient cohort.

The ACPGBI consensus [6] recommends surgical intervention as definitive management of sigmoid volvulus; however, the timing of this is uncertain–a poll conducted by the Association of Surgeons of Great Britain and Northern Ireland showed that 75% of surgeons would consider surgery, but only 23% would perform this on index admission. In addition, 2% stated they avoid surgery completely [16]. This variation in treatment opinions reiterates the fact that treatment of sigmoid volvulus should be determined with a holistic approach, with shared decision‐making between patient, their families/carers and their clinician. The pursuit of further research is not only important to ensure that the most appropriate outcomes are measured, but to provide an evidence base on which clinicians can base their decision‐making.

There are several limitations to the study. The overall number of patients included was relatively low and therefore there may be an element of self‐selection bias, with patients who had either an extremely positive or negative experience being motivated to attend. This is a known limitation of PPI work and our group did have a broad variety of patients and their representatives and tried to maximise participation through our hybrid approach online and in‐person. It is recognised that too many participants can dilute engagement [17]. The research team in attendance at the PPI event was also relatively large (7 people across two sites) compared to the numbers of patients and carers in attendance. We do not believe that this influenced the group dynamics as the discussions were led at each site by a single researcher (NH and SM) with the others in attendance there to help facilitate the discussions and to take notes. Our group also had a large proportion who had undergone surgery; this may be due to them being fitter and therefore more able to physically attend than those patients who have been managed without surgery. Our group was also comprised of white British patients, with no representation from other ethnic or cultural groups. As there is currently limited UK data on sigmoid volvulus it is not possible to assess whether our group is representative of the UK population who develop/suffer with sigmoid volvulus. Many patients with sigmoid volvulus are living with frailty and multi‐morbidity and were unable to attend, and although we did explore the use of attendance of an individual online, our experience of previous PPI work has demonstrated the importance of a face‐to‐face facilitator. Furthermore, many patients with sigmoid volvulus have died‐either due to volvulus or other causes, which could have resulted in survivorship bias. Our participants voiced that it was important to include relatives/carers of those that had died and although challenging, we aim to include those groups in our future work [18].

## CONCLUSION

Sigmoid volvulus is a common surgical emergency presentation, which significantly impacts patients and their relatives/carers. The key findings of this PPI day, including the impact of clinical decision‐making on quality of life after a diagnosis of sigmoid volvulus and its impact on wider family, will be used to inform future study designs to improve our understanding of the treatment and outcomes for patients with sigmoid volvulus.

## AUTHOR CONTRIBUTIONS

**Zak Shehata:** Writing – original draft; investigation; formal analysis. **Nick Heywood:** Conceptualization; investigation; writing – review and editing; supervision. **Sue Blackwell:** Writing – original draft; investigation; formal analysis. **Susan Moug:** Investigation; writing – review and editing. **Jennifer Law:** Investigation; writing – review and editing; supervision. **Carly Bisset:** Investigation; writing – review and editing. **Hwei Jene Ng:** Investigation; writing – review and editing.

## FUNDING INFORMATION

This work was supported by a grant from Bowel Research UK. The funder has not had any input into the design or delivery of this study.

## CONFLICT OF INTEREST STATEMENT

The authors declare no conflicts of interest.

## Data Availability

Data sharing not applicable to this article as no datasets were generated or analysed during the current study.
